# Rational Design of Cu@Pd Core–Shell Nanostructures via Galvanic Replacement for Dual Electrochemical Applications: Hydrogen Evolution and Nitrate Reduction Reactions

**DOI:** 10.3390/molecules30204062

**Published:** 2025-10-12

**Authors:** Bommireddy Naveen, Sang-Wha Lee

**Affiliations:** Department of Chemical and Biological Engineering, Gachon University, 1342 Seongnam-daero, Seongnam-si 13120, Republic of Korea; naveenb@gachon.ac.kr

**Keywords:** electrodeposition, galvanic replacement, hydrogen evolution, nitrate reduction reaction, cyclic voltammetry, Tafel analysis

## Abstract

Developing bifunctional electrocatalysts that simultaneously enable green hydrogen production and water purification is essential for advancing sustainable energy and environmental technologies. In this study, we present Cu@Pd core–shell nanostructures fabricated through template-assisted electrodeposition of Cu, followed by galvanic Pd modification on pyrolytic graphite electrodes (PGEs). The optimised catalyst exhibited superior hydrogen evolution reaction (HER) activity, with an onset potential of 70 mV, a low Tafel slope of 33 mV dec^−1^ and excellent stability during prolonged HER operation. In addition to hydrogen evolution, Cu@Pd/PGE shows significantly enhanced nitrate reduction reaction (NRR) activity compared to Cu/PGE in both alkaline and neutral conditions. Under ideal conditions, the catalyst achieved 60% nitrate removal with high selectivity towards ammonia and minimal nitrite formation, emphasising its superior performance. This enhanced bifunctionality arises from the synergistic Cu–Pd interface, facilitating efficient nitrate adsorption and selective hydrogenation. Despite their high catalytic activity for both HER and NRR, the Cu@Pd nanostructures could often emerge as a versatile platform for integration into sustainable hydrogen production and an effective denitrification process.

## 1. Introduction

The increasing global demand for sustainable energy and environmental solutions has prompted extensive research into electrocatalysis, particularly for processes related to clean energy production and pollution remediation. In this context, hydrogen evolution reaction (HER) and nitrate reduction reaction (NO_3_^−^RR or NRR), have emerged as important electrochemical processes, due to their roles in carbon-neutral hydrogen generation and decentralised ammonia synthesis, respectively [1]. Despite this significance, the development of bifunctional electrocatalysts capable of mediating these diverse electrochemical transformations holds significant potential towards sustainable hydrogen production and environmental technologies.

Nitrate contamination represents a significant environmental issue in both developed and developing regions, primarily due to industrial discharges and excessive fertiliser usage. Elevated nitrate levels in drinking water frequently exceed the WHO-recommended limit of 50 mg/L, resulting in adverse health effects such as methemoglobinemia or blue baby syndrome, particularly in pregnant women and infants [2]. Conventional nitrate removal technologies, including ion exchange [3], reverse osmosis [4] and bacterial nitrification [5], are often associated with high operational costs and a tedious technological process, making NRR a cleaner and sustainable alternative. NRR proceeds through a sequence of adsorption intermediates (*NO_3_, *NO_2_, *NH_2_OH, *NH_3,_ etc.) and involves a cascade of multi-electron and multi-proton reactions, resulting in either benign N_2_ or value-added compounds like ammonia. Various metallic catalysts with high catalytic activity, such as Bi, Ru, Ag, Pd, Pt, Cu and Fe, are being used for the electrochemical reduction of nitrate [6,7,8,9,10]. Despite the lower *NO_3_ dissociation barrier, Pd is expected to have faster NO_3_^−^ reduction kinetics. Pd also facilitates the *N-N coupling, a crucial step in forming N_2_. Additionally, palladium undergoes surface oxidation to form Pd-O and/or Pd-OH species that can act as active centres for specific adsorption of nitrate, supported by its high adsorption energy (−3.22 eV) [11].

Interestingly, the surface reactivity that enables Pd to be effective for NRR also significantly influences the hydrogen evolution reaction (HER), a crucial process in water splitting. The adsorption and desorption dynamics of hydrogen intermediates regulate HER. A volcano plot based on the hydrogen binding energy (ΔG_H*) is typically used to assess these dynamics [12]. Due to optimal binding energy (~0 V), platinum and Pt-based materials exhibit minimal overpotentials (η) and high exchange current densities, placing them at the apex of the HER volcano plot [13]. However, their high cost and restricted availability impede their large-scale applications. Consequently, developing non-Pt catalysts that exhibit high stability and activity remains a significant challenge in electrochemical water splitting [14]. Several transition metal-based catalysts, such as Rh, Pd, Fe, Ni and Re have been the focal points of research on HER [1,14,15]. Specifically, Pd-based nanostructures have emerged as promising materials in various electrochemical transformations, owing to their high catalytic activity and strong affinity for hydrogen-related processes [16,17,18,19]. Palladium with a face-centered cubic lattice exhibits high current densities towards HER due to its hollow site [20]. DFT studies further revealed that the Pd (111) facet has a high integral adsorption energy of 81 KJ mol^−1^ of H_2_ and displays high HER at low overpotentials [20].

Pd exhibits strong hydrogen adsorption at very low cathodic potentials, enabling key HER pathways (Volmer–Heyrovsky or Volmer–Tafel), but may potentially impede the adsorption of nitrogen intermediates in the NRR, underscoring the complexity of the catalyst’s selectivity. Therefore, careful design of the catalyst is essential, especially when both HER and NRR operate within similar potential ranges. Core–shell nanostructures, featuring a thin layer of active material (Pd) and an abundant core metal such as copper, have emerged as a promising strategy. These catalytic nanostructures often demonstrate high synergistic effects without compromising their catalytic efficiency. The copper core can modulate the Pd surface and potentially optimise the adsorption energetics of intermediates in both NRR and HER. In nitrate reduction, the copper core electronically modulates the surface palladium, encouraging the adsorption and activation of *NO_3_^−^ and *NO_2_^−^ enabling efficient catalysis. For HER, copper can shift the d-band centre of Pd and help in reducing the overpotential.

In the present study, we report the facile fabrication of Cu@Pd core–shell nanostructures on cost-effective pencil graphite electrodes using electrodeposition and galvanic replacement. We explored their potential as bifunctional electrocatalysts for both hydrogen evolution and nitrate reduction reactions. Unlike conventional bifunctional catalysts, which often suffer from instability, high cost and lower selectivity towards desirable products, the Cu@Pd system not only shows Pt-like activity for HER but also achieves significant nitrate conversion with excellent selectivity towards ammonia. This suggests the potential of the catalyst as a durable and scalable platform for clean energy generation and environmental remediation.

## 2. Results and Discussion

### 2.1. Material Characterisation

Copper nanostructures were electrochemically deposited onto a highly oriented polymer carbon electrode using an aqueous solution containing CuCl_2_, KCl and SDS at a concentration just above its critical micellar concentration (CMC), in accordance with our previous reports [21,22]. The presence of SDS alters the local deposition environment by changing the deposition overpotentials, which in turn affects the nucleation and growth mechanisms. Furthermore, SDS significantly influences the diffusion coefficient of Cu^2+^ ions through micellar-mediated transport, facilitating the formation of a distinct dendritic morphology [22]. Figure S1a,b represents the SEM analysis of the Cu/PGE, indicating that the resulting nanostructures exhibit dendritic morphology characterised by high surface area and porosity. Palladium modification could be achieved using galvanic replacement reaction (GRR), since the standard reduction potential of palladium (Pd^2+^/Pd = 0.95 V) is much higher than that of copper (Cu^2+^/Cu = 0.34 V). Therefore, palladium could easily replace copper on the graphite electrode, resulting in the formation of a monolayer and/or several layers, as expressed by the following equation:

$${Cu}_{s}^{0}/PGE+{Pd}_{aq}^{2+}\to Cu@Pd/PGE+{Cu}_{aq}^{2+}$$

The replacement reaction was monitored using open-circuit potential (OCP) measurements. The OCP profile, as illustrated in Figure S2a, shows three characteristic stages: (I) an initial region ~0 V corresponding to the bare Cu surface; (II) a gradual increase in the potential up to +0.1 V could be attributed to Pd nucleation, accompanied by copper oxidation and subsequent dissolution; and (III) a sudden jump in the potential (observed at +0.1 V) which subsequent plateau at +0.48 V, is due to rapid nucleus growth and filling of adjacent voids to obtain Pd^2+/^Pd equilibrium [23].

**Figure 1:**
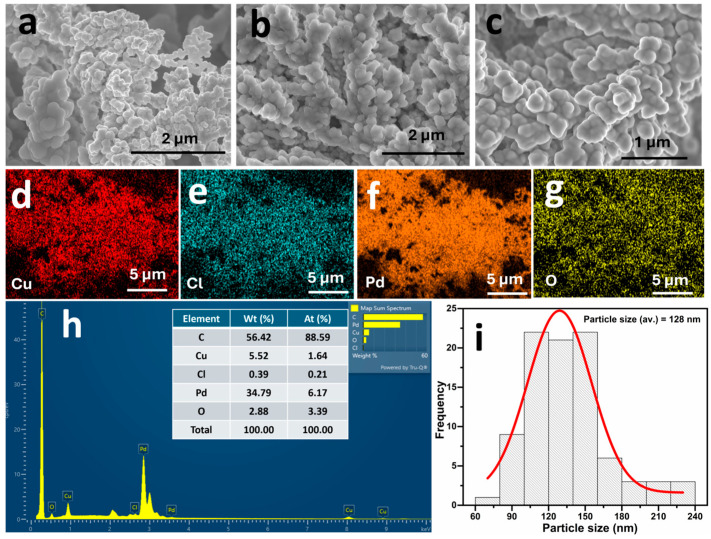
SEM micrographs of Cu@Pd/PGE at various magnifications (**a**–**c**), elemental mapping along with the EDX analysis of Cu@Pd/PGE (**d**–**h**) and a histogram showing the particle size distribution of Cu@Pd/PGE (**i**).

Figure 1a–c displays the FE-SEM micrographs of Cu@Pd core–shell nanostructures that formed after the galvanic replacement. The SEM images reveal that the dendritic porous copper nanostructures became slightly coarsened after palladium modification; however, they largely retain their copper adatom morphology. The surface appears more granular and compact following GRR, suggesting that palladium nucleation primarily occurred on the outer layers of copper. Furthermore, the absence of significant structural changes suggests the formation of a few Pd layers, aligning with previous reports, as bulk changes could significantly alter morphology. Figure 1d–g represents the elemental mapping corresponding to Cu, Cl, Pd and O, confirming an even distribution of copper and palladium across the electrode surface. Chlorine originates from residual chloride in the CuCl_2_ and PdCl_2_ precursors, while oxygen likely results from surface oxides (Cu_2_O, CuO and PdO) formed during electrochemical treatment. EDX mapping, as shown in Figure 1h, suggests that palladium content on the Cu@Pd electrode is 34.79% by weight and 6.17% by atomic ratio. The relatively high weight ratio of Pd to Cu (34.79% to 5.52%), coupled with the low atomic percentage of Pd, further supports that palladium forms a thin layer on the copper core. Additionally, the particle size distribution histogram in Figure 1i reveals a narrow distribution with the average particle size of 128 nm.

**Figure 2:**
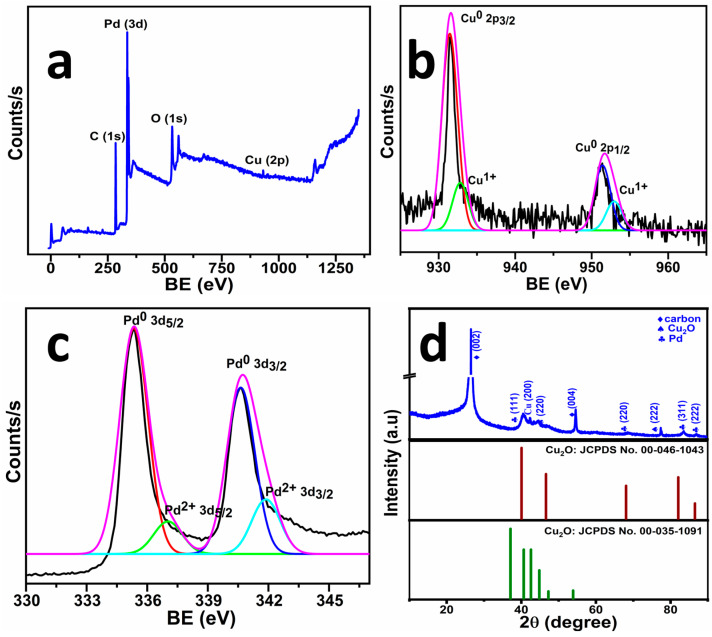
XPS survey spectra of Cu@Pd/PGE (**a**), deconvoluted spectra of Cu 2p (**b**) and Pd 3d (**c**). XRD patterns of Cu@Pd core–shell nanostructures (**d**).

XPS and XRD analyses were performed to determine the precise chemical composition and crystallinity of the Cu@Pd-modified graphite electrode. The survey scan spectra in Figure 2a confirm the presence of characteristic peaks corresponding to C, Cu, O and Pd elements. High-resolution elemental spectra of copper, as shown in Figure 2b, reveal two characteristic peaks at 931.5 eV (Cu 2p_3/2_) and 951.3 eV (Cu 2p_1/2_), which are typically attributed to Cu^0^ [24,25]. Deconvolution of the Cu spectrum further reveals additional peaks at 933.3 eV and 953.5 eV, corresponding to Cu^+^ species, associated with the formation of Cu_2_O [26]. As illustrated in Figure 2c, Pd exhibit two sharp peaks at binding energies of 335.3 eV and 340.6 eV, corresponding to Pd^0^ 3d_5/2_ and Pd^0^ 3d_3/2_, respectively [16,27]. Additionally, two peaks at 337 eV and 341.9 eV are attributed to Pd^2+^ species formed by the inevitable surface oxidation upon exposure to atmospheric conditions [28]. The O 1s spectrum was deconvoluted using Gaussian functions and determined to peaks (1), (2) and (3) with binding energies of 529.5, 532.1 and 533.7 eV, respectively. The lowest binding energy Peak (1) could possibly be attributed to the adsorption of O^2−^ ions associated with CuO and/or PdO phases. Peak (2) could be correlated to defect-related oxygen vacancies, whereas peak (3) can be assigned to surface OH^−^ ions in oxygen-lacking regions [29]. The overall atomic composition of the Cu@Pd sample, estimated from XPS, was Cu:Pd:O = 1:1.1:2.3.

The XRD pattern in Figure 2d confirms the crystalline nature of the prepared Cu@Pd nanostructures. Diffraction peaks at 2θ values of 40.4°, 62.8°, 82.9° and 86.6° correspond to the (111), (220), (311) and (222) lattice planes of palladium’s FCC structure [28]. Additionally, intense diffraction peaks were observed at 2θ values of 42.6° and 44.6° corresponding to Cu (200) and Cu_2_O (220) phases, suggesting surface oxidation of copper during the replacement reaction [30]. According to the literature, the intensity of metallic copper peaks generally decreases with the plating of another metal, such as palladium, which could possibly impact the Cu crystal facets [30]. Due to the high standard reduction potential of palladium, copper is expected to undergo partial oxidation in the aqueous electrolyte during GRR. These results, together with XPS and FE-SEM, suggest the formation of a mixture of oxides on the electrode surface.

The electrochemical surface area (ECSA) of the prepared electrodes was calculated using the following equation:

ECSA = C_dl_/C_s_

To determine the double-layer capacitance (C_dl_), cyclic voltammetry curves were measured in a potential window where the Faradaic process was nearly absent at different scan speeds (10–100 mV/s), as shown in Figure S3. A linear relationship was obtained between the current density (ΔJ = J_a_ − J_c_) at a set potential against scan rate, and the slope of the line, C_dl_, is calculated to be 689 µF. The standard capacitance (C_s_) of the metal electrodes in the acid electrolyte was 20 µF/cm^2^ [31,32]. The roughness factor of the fabricated electrode was estimated to be 34.45.

### 2.2. Hydrogen Evolution Reaction (HER)

**Figure 3:**
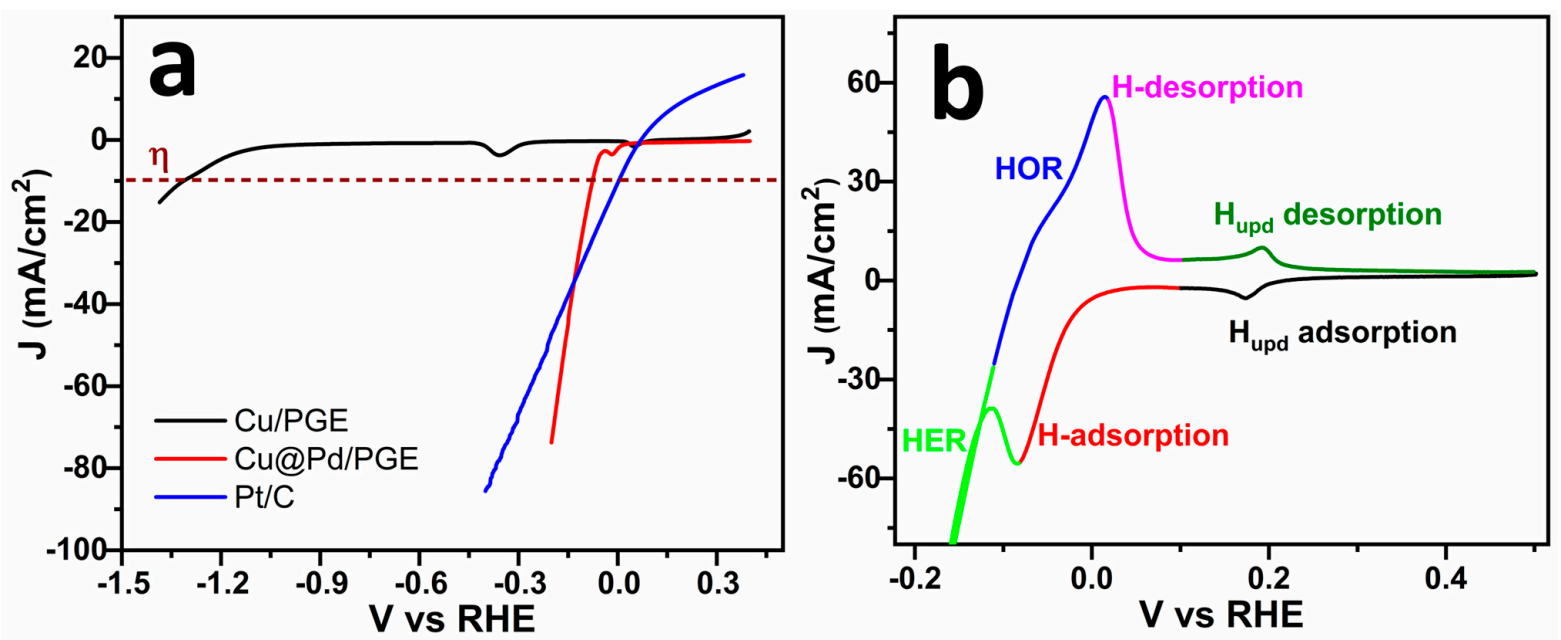
LSV curves of Cu/PGE, Cu@Pd/PGE, and commercial Pt/C (**a**), cyclic voltammogram showing hydrogen probing regions on the Cu@Pd/PGE electrode (**b**): All experiments were conducted in 0.5 M H_2_SO_4_ at a scan rate of 50 mV/s.

The surface characteristics and hydrogen evolution activity of the fabricated Cu@Pd core–shell nanostructures were evaluated in 0.5 M H_2_SO_4_ solution. Figure 3a illustrates the HER polarisation curves for Cu/PGE, Cu@Pd/PGE, and commercial Pt/C. The bare Cu/PGE electrode exhibited poor catalytic activity, requiring a high overpotential (η) of −1.30 V to achieve noticeable HER current response. In contrast, modification with Pd significantly enhanced the performance: the Cu@Pd/PGE requires an overpotential of 70 mV to achieve a standard current of 10 mA/cm^2^ and delivered 77 mA/cm^2^ at −150 mV. Notably, this activity exceeded that of commercial Pt/C, which achieved 35 mA/cm^2^ at the same overpotential. The superior catalytic behaviour of Cu@Pd/PGE can be attributed to the synergistic interactions between the conductive Cu core and the catalytically active Pd shell, which optimises the hydrogen adsorption free energy (ΔG_H*), to expedite the Volmer and Heyrovsky reaction steps.

Further mechanistic insights were obtained from cyclic voltammetry profiles, which highlight hydrogen adsorption–desorption characteristics, as depicted in Figure 3b. Symmetrical cathodic and anodic peaks at 0.17 V and 0.21 V are attributed to underpotential deposition (UPD) of hydrogen adsorption (UPD-H_ads_) and hydrogen desorption (UPD-H_des_), respectively, which are commenced at potentials positive to H^+^/H_2_, typically referred to as the Volmer step [33,34]. The calculated ΔE_p_ for the UPD-H is 38 mV, which is very close to the reported value of 35 mV. At increasingly negative potentials, hydrogen will undergo specific absorption into the Pd subsurface, forming Pd hydrides, while hydrogen evolution occurs at higher negative potentials [35]. The reversible hydrogen desorption and HOR peaks indicate that the prepared nanostructures exhibit purely adsorptive behaviour.

To optimise the palladium coating process, Cu@Pd electrocatalysts were fabricated with varied copper deposition times (3, 5, and 7 min), prior to Pd shell formation, enabling a systematic evaluation of their HER performances, as presented in the LSV profiles (Figure S4a). As observed, the Cu@Pd-7 min displayed the highest current density, suggesting optimal coverage and availability of active sites. Stability assessments further corroborated this result: cycling stability at −0.4 V, as presented in Figure S4b, reveals that the Cu@Pd-3 min electrode retained 106% of its initial value after 1000 cycles, whereas the Cu@Pd-7 min electrode displayed high catalytic activity and increased progressively with cycling. Further long-term stability studies were conducted using amperometric and chronopotentiometric measurements, as shown in Figure S4c,d. The Cu@Pd-7 min electrode displayed stable HER activity at an overpotential of 100 mV over prolonged operation. Similarly, chronopotentiometric studies suggest that the electrode exhibited less than 2% potential drift at a constant current of 50 mA/cm^2^. Owing to its superior performance, the Cu@Pd-7 min electrode was selected for further studies.

**Figure 4:**
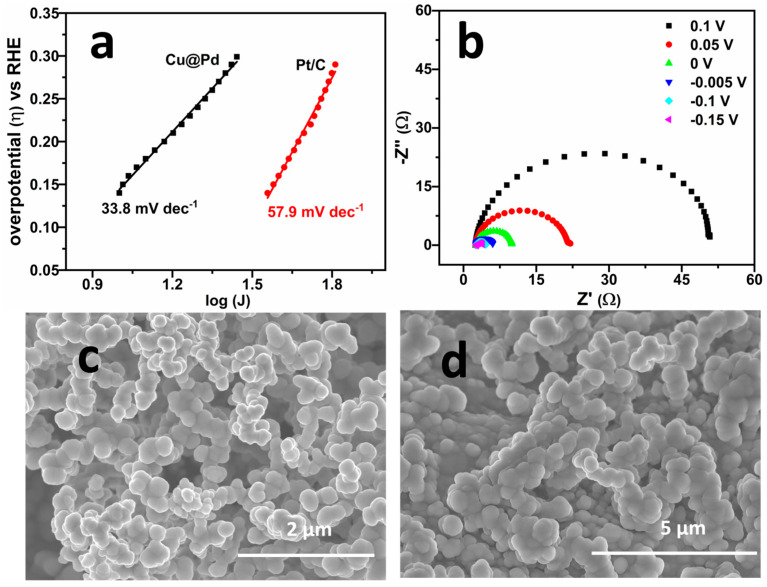
Tafel slope analysis for the Cu@Pd/PGE and commercial Pt/C for the hydrogen evolution reaction (**a**), electrochemical impedance analysis of Cu@Pd/PGE towards HER (**b**) and SEM images of Cu@Pd/PGE nanostructures after stability studies (**c**,**d**).

To gain further insights into the reaction kinetics, Tafel slope and electrochemical impedance measurements were conducted, and the results are presented in Figure 4a,b. The Tafel plot for Cu@Pd/PGE shows a slope of 33.8 mV dec^−1^, significantly lower than that of commercial Pt/C (57.9 mV dec^−1^), thereby facilitating electron transfer for HER over Cu@Pd/PGE. A Tafel slope close to 30 mV/dec is indicative of the Volmer-Tafel pathway, where the rate-determining step is the recombination of adsorbed hydrogen atoms (2H* → H_2_), consistent with Pd-based nanomaterials [36,37]. Further EIS results show that Nyquist plots are characterised by a decrease in the radius of the semicircle with increasingly negative potentials. The EIS data were fitted with Randle’s circuit to obtain the charge transfer resistance, and the fitted values are summarised in Table S1. The table revealed that Cu@Pd/PGE had significantly lower impedance at higher potentials, as seen by the shortest semicircle and correspondingly lowest R_ct_ values. The significant decrease in the impedance enables HER kinetics to occur much faster, thereby enhancing the rate of HER.

The morphological stability of the catalyst was subsequently examined by SEM following amperometric stability tests, as illustrated in Figure 4c,d. The fresh Cu@Pd electrode features a dendritic, nanocluster-rich structure that offers a high surface area and promotes efficient mass transport, thereby enhancing catalytic activity. After prolonged catalytic operation, SEM images reveal only minor smoothing of the dendritic branches, attributed to HER and bubble dynamics, while the core dendritic structure remains intact. This structural durability directly correlates with the observed long-term electrochemical stability, emphasising that the Cu@Pd catalyst combines high activity with strong physical stability. Table 1 provides a comparison of different Pd-loaded composites for HER. Among the examined materials, Pd-CNx has the lowest overpotential and Tafel slope, likely due to the synergistic effect of Pd with graphitic carbon nitride, which enhances electron transfer. The table shows that the prepared Cu@Pd core–shell nanostructures outperform other palladium composites in terms of overpotential, Tafel slope and retention activity.

### 2.3. Nitrate Reduction Reaction (NRR)

In the absence of nitrate, the LSV profile of Cu/PGE, as shown in Figure S5a, displayed a cathodic peak at higher negative potentials, which could be ascribed to the HER. Upon the addition of nitrate, a distinct cathodic peak appeared at −1.17 V, indicating nitrate reduction, consistent with earlier reports for Cu single crystals and Cu-based catalysts [22,44]. In order to investigate the kinetics of the reaction, CVs were recorded at different scan rates (10–100 mV/s), as shown in Figure S5b. The peak currents increased steadily with negligible peak shifts, suggesting a stable electrode-electrolyte interface. Moreover, the peak current exhibited a linear dependence on the square root of the scan rate, as displayed in Figure S5c, indicating that the nitrate reduction process on Cu/PGE is mainly diffusion-controlled. According to the literature, the initial product of nitrate reduction is nitrate [44]. In our studies, the small cathodic peak at −0.92 V on the copper electrode is likely associated with the formation of nitrite.

**Figure 5:**
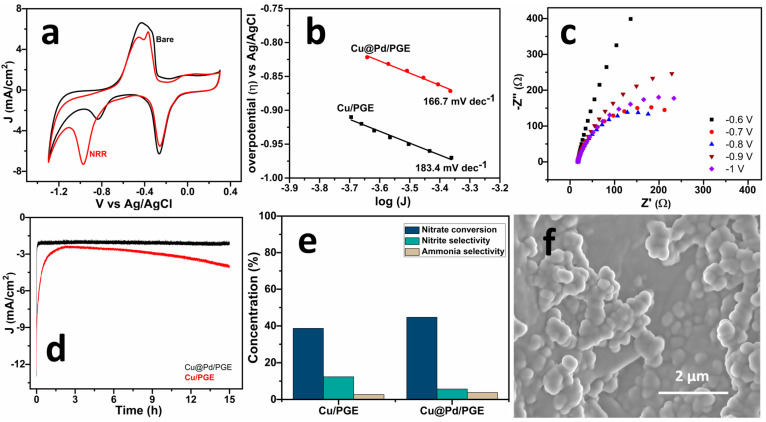
CV of Cu@Pd/PGE in the absence and presence of nitrate (**a**), Tafel slope analysis for the Cu@Pd/PGE and Cu/PGE towards NRR (**b**), impedance measurements recorded at various potentials for Cu@Pd/PGE (**c**), amperometric stability of Cu/PGE at −1.2 V and Cu@Pd/PGE at 1.0 V towards NRR (**d**), estimation of nitrate converted and the formation of nitrite and ammonia after catalysis (**e**) and SEM micrograph of Cu@Pd/PGE after catalysis (**f**). All the studies were carried out in 10 mM of NaNO_3_ with 0.1 M KOH as the supporting electrolyte.

The catalytic performance of the Cu@Pd/PGE electrode towards NRR was subsequently evaluated and compared with Cu/PGE. As illustrated in Figure 5a, the CV of Cu@Pd exhibits high HER in the absence of nitrate and a pronounced peak at −0.96 V, which is substantially more positive than that of Cu/PGE (−1.15 V) with the addition of nitrate. This shift underscores the beneficial role of the Pd overlayer in facilitating nitrate activation at lower overpotentials while suppressing the competing HER in alkaline environments [45]. Tafel slope analysis, as shown in Figure 5b, further corroborates this enhancement, with Cu@Pd/PGE exhibiting a slope of 166.7 mV dec^−1^ compared to 183.4 mV dec^−1^ for Cu/PGE, suggesting better electron transfer kinetics, which is often attributed to hydrogenation kinetics on Pd-surfaces [46]. Figure 5c presents the Nyquist plots of the EIS of Cu@Pd/PGE at different applied DC potentials, revealing that the R_ct_ value decreases at higher negative potentials and reaches its lowest value at −1.0 V, aligning with the CV results discussed earlier.

Chronoamperometry was employed to evaluate the long-term activity of the catalyst and to determine the products formed after catalysis, as shown in Figure 5d. For Cu/PGE, a gradual increase in the current was observed at −1.2 V, which could be attributed to surface restructuring of the Cu surface [47]. This could possibly increase the active electrochemical surface area and allow parasitic HER, thereby reducing the overall selectivity for nitrate conversion [48]. In contrast, Cu@Pd/PGE maintained a stable current at −1.0 V, suggesting that Pd coating mitigates surface reorganisation and suppresses HER [49]. The product selectivity profiles displayed in Figure 5e further validate the superiority of Cu@Pd/PGE in nitrate conversion. Although the total nitrate conversion over Cu/PGE and Cu@Pd/PGE was comparable, the product distribution varied significantly. Cu/PGE shows high selectivity towards the formation of nitrite, suggesting a dominant 2-electron transfer reaction with minimal ammonia generation. Conversely, Cu@Pd/PGE exhibit high selectivity towards the formation of ammonia with a complete 8-electron transfer. This improved selectivity towards ammonia could be attributed to the successive hydrogenation of *NO_2_ and *NO intermediates to NH_3_ over Pd active sites, in accordance with the previously reported literature [45,49]. Post-reaction SEM analysis (Figure 5f) revealed that the dendritic morphology of Cu@Pd was largely retained, further highlighting the structural robustness of the electrode under prolonged operation.

**Figure 6:**
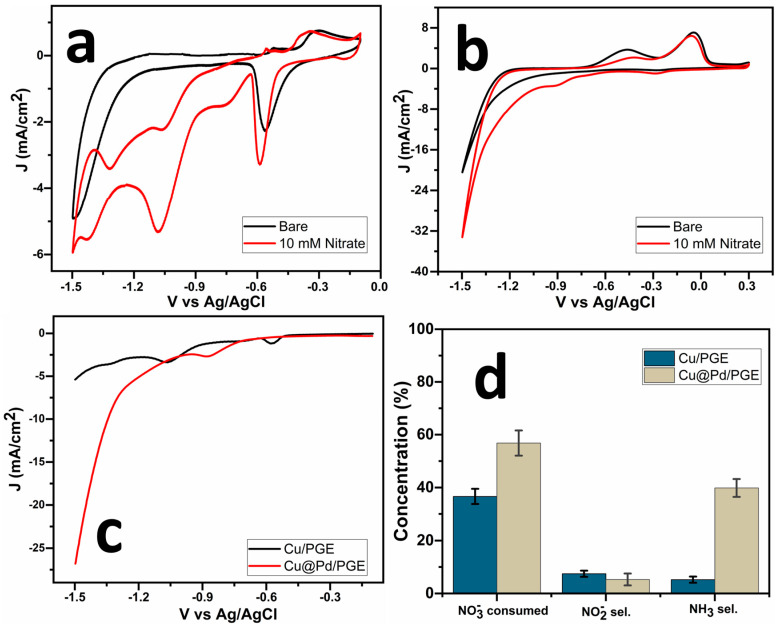
CV profiles of Cu/PGE (**a**) and Cu@Pd/PGE (**b**) in the absence and presence of sodium nitrate in 0.05 M Na_2_SO_4_. Linear sweep voltammograms of Cu/PGE and Cu@Pd/PGE in 10 mM of nitrate (**c**) and product distribution of Cu/PGE and Cu@Pd/PGE towards nitrate electrocatalysis in 0.05 M 5 M Na_2_SO_4_ (**d**).

Following the evaluation in alkaline media, the electrocatalysts were further studied in neutral media (0.05 M Na_2_SO_4_, 6.28 pH) to assess their NRR activity. The CV of Cu/PGE in the presence of nitrate, as shown in Figure 6a, revealed two broad cathodic peaks at −0.77 V and at −1.1 V vs. Ag/AgCl, respectively, which can be ascribed to the stepwise reduction of nitrate to nitrite, followed by the subsequent reduction of nitrite into other nitrogen species such as hydroxylamine or ammonia. This observation aligns well with the earlier reports over the Cu electrodes in alkaline and neutral media, in which nitrate undergoes a diffusion-controlled reaction [22,44,50].

In contrast, the palladium-modified Cu/PGE, as exhibited in Figure 6b, displays a shoulder peak at −0.83 V vs. Ag/AgCl, typically corresponding to the onset potential for nitrate reduction, aligning with the reported literature for ordered Pd-Cu electrocatalysts [49]. As illustrated in Figure 6c, Cu@Pd/PGE demonstrates a superior current density throughout the potential range compared to Cu/PGE, surpassing HER in the presence of nitrate. It has been reported that the increase in hydrogen population on the electrode surface promotes the production of ammonia [51]. This suggests a significant improvement in electron-transfer kinetics and enhances catalytic efficiency towards NRR with the incorporation of Pd. The product analysis presented in Figure 6d further supports these observations. Although Cu/PGE achieves moderate nitrate conversion, producing mainly nitrate, it highlights its limited ability to facilitate the multi-electron pathway after the initial reduction step. Conversely, Cu@Pd/PGE converts ~60% of nitrate with ammonia as the primary product and minimal nitrite formation, thereby affirming the role of Pd in stabilising adsorbed nitrate and reducing the energy barrier for further hydrogenation.

The catalytic efficiency of Cu@Pd/PGE is higher in neutral media (pH 6.8) than in alkaline conditions, as demonstrated by improved nitrate reduction efficiency, lower nitrite formation, and greater catalytic stability. Table 2 shows that the performance of Cu@Pd/PGE matches or surpasses that of many recently reported electrocatalysts for nitrate reduction.

## 3. Experimental

**Scheme 1:**
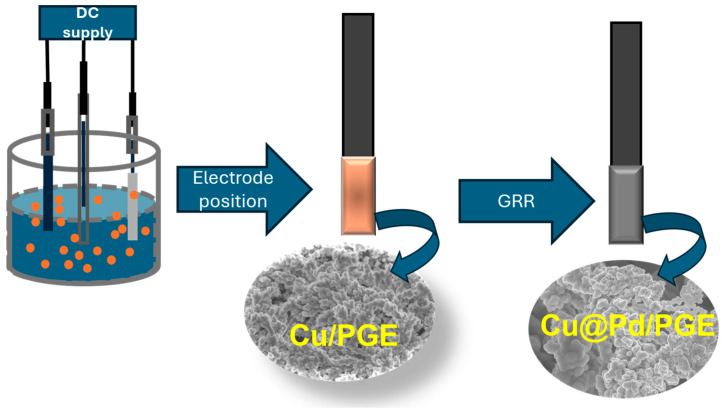
Schematic representation of the preparation of Cu@Pd core–shell nanostructures on PGE substrate.

In this research, the deposition of Cu@Pd core–shell nanostructures on a pencil graphite electrode (PGE) was prepared by employing a Zive SP1 potentiostat (ZIVELAB, Seoul, Republic of Korea). A highly oriented polymer graphite electrode (PGE, Faber-Castell, Stein, Germany, 0.7 mm HB) was used as the primary substrate for surface modifications. Platinum wire was used as the auxiliary electrode, and a saturated calomel electrode (SCE, Sat. KCl) was used as the reference electrode for electrodeposition. Cu@Pd nanostructures were prepared in accordance with our prior investigations [21,22]. In brief, copper deposits were prepared by applying a constant potential of −0.7 V vs. SCE in a solution containing 15 mM CuCl_2_, 0.1 M KCl and 14 mM sodium dodecyl sulphate (SDS) for different deposition intervals such as 3, 5 and 7 min. Here, SDS was used as a soft template to alter the nucleation mechanism and physical arrangement of the electrodeposited nanostructures (Cu/PGE). Pd modification was achieved through a galvanic replacement reaction using PdCl_2_ solution in 0.1 M perchloric acid as the supporting electrolyte, and Cu/PGE as the working electrode. The overall reaction was monitored using the open-circuit potential technique as presented in Scheme 1.

The prepared Cu@Pd core–shell nanostructures were rinsed with distilled water to remove loosely bounded particles before proceeding for further examination. For HER 0.5 M H_2_SO_4_ was chosen as the electrolyte, while nitrate reduction tests were conducted in two different electrolytes 0.1 M KOH and 0.05 M Na_2_SO_4_ in a 25 mL solution with 15 mM NaNO_3_. During NRR, when 0.1 M KOH was used as the electrolyte, the pH was 11.6; in contrast, with 0.05 M Na_2_SO_4_, the pH was 6.28. Cyclic voltammograms were performed at a 50 mV/s scan rate, while linear sweep voltammograms were conducted at a 10 mV/s scan rate against an Ag/AgCl reference electrode. Impedance measurements were conducted within a frequency range of 100 mHz to 100 kHz with an applied AC amplitude of 5 mV. All the cathode potentials in the study were converted to reversible hydrogen electrode (RHE) using the following equation.

E_RHE_ = E_Ag/AgCl_ + 0.198 V + (0.059 pH)

The morphology of the fabricated nanostructures was examined using a field-emission scanning electron microscope (FE-SEM, HITACHI SU8600, Hitachi High-Tech corporation, Seoul, Republic of Korea) at an accelerating voltage of 5 kV at the Smart Materials Research Centre for IoT at Gachon University. X-ray diffraction (XRD) analysis was performed using a Bruker D8 (Bruker, Berlin, Germany) Advance diffractometer with Cu Kα irradiation (λ = 0.154184 nm) to determine the phase and orientation of the deposited films. X-ray photoelectron spectroscopy (XPS) measurements were performed on the catalyst surface using a Thermo Fisher K-Alpha spectrometer (Thermo Fisher Scientific, Seoul, Republic of Korea), with Al Kα radiation (1486.6 eV) for excitation, to determine the valence states.

The ultraviolet-visible (UV-Vis) spectrophotometer was used to determine the ion concentrations of pre- and post-electrolyte samples after diluting them to the proper concentrations to match the calibration curves. The concentration of nitrate and nitrite in the electrolyte was determined directly. An aliquot of the reaction electrolyte was taken and diluted to 5 mL with deionised water. The absorbance of the diluted solution was then recorded. Ammonia quantification was conducted using the indophenol blue method. For this, 3 mL of the reaction mixture was mixed with 500 µL of phenol nitroprusside solution (P6694, Sigma-Aldrich, St. Louis, MO, USA) and 500 µL of alkaline hypochlorite solution (A1727, Sigma-Aldrich, St. Louis, MO, USA). The UV-Vis spectra were recorded after 30 min of incubation at room temperature in dark conditions.

## 4. Conclusions

In conclusion, Cu@Pd core–shell nanostructures were prepared via template-assisted electrodeposition of Cu, followed by galvanic replacement for Pd modification. The prepared electrodes exhibit outstanding bifunctional activity towards both the HER and NRR. The electrode prepared with 7 min of copper deposition delivered superior HER performance, marked by a low onset potential of 70 mV, a small Tafel slope of 33 mV dec^−1^, and remarkable stability over 1000 potential cycles. Long-term durability tests at −0.1 V (amperometry) and at −50 mA/cm^2^ (galvanostatic) revealed only ~2% decay after 30 h. For NRR, the Cu@Pd/PGE electrode achieved ~60% nitrate removal in neutral 0.05 M Na_2_SO_4_ with high selectivity for ammonia, outperforming the bare Cu/PGE electrode. Together with efficient HER activity and selective nitrate conversion, the Cu@Pd nanostructures could emerge as versatile electrocatalysts for sustainable hydrogen production and water purification.

## Figures and Tables

**Table 1:** Comparison of HER performance of different palladium-loaded electrocatalysts towards hydrogen evolution reaction in acidic medium.

Catalyst	Electrolyte	Overpotential (η) (mV vs. RHE)	Tafel Slope (mV dec^−1^)	Ref.
Pd/MOF	0.5 M H_2_SO_4_	105	85	[38]
Pd-CNx	0.5 M H_2_SO_4_	55	35	[39]
n-Pd@NDCDs	0.5 M H_2_SO_4_	291	135	[40]
n-PdCu@NDCDs	0.5 M H_2_SO_4_	115	-	[41]
MoSe_2_/Pd	0.5 M H_2_SO_4_	231	69	[42]
Pd/WS_2_	0.5 M H_2_SO_4_	130	82.4	[43]
(NiPd)_17_Se_15_	0.5 M H_2_SO_4_	197	91.5	[43]
Cu@Pd/PGE	0.5 M H_2_SO_4_	150	33.8	Present study

**Table 2:** Performance comparison of different electrocatalysts towards nitrate reduction reaction.

Catalyst	Methodology	Electrolyte	Efficiency	Ref
Pd_40_Cu_60_/SS	Electrodeposition	50 mg/L NO_3_-N in 0.05 M NaClO_4_	Potential = −0.3 V, N_2_ selectivity = 40%	[52]
Pd_20_Cu_80_/Cu	Ball milling	0.1 M NO_3_-N in 0.1 M NaOH	NH_3_ selectivity = 61% N_2_ selectivity = 39%	[53]
Pd_80_Cu_20_/NiAl	Coprecipitation	3 mM NO_3_-N in 1 M KOH	Sorption of NO_3_-N achieved 2 mg/g at 1.0 V, removal rate = 68%	[54]
Bi_10_Cu_90_/Cu	Electrodeposition	100 mg/L NO_3_-N in 0.1 M Na_2_SO_4_	Removal rate = 87.5% at 6 mA/cm^2^	[55]
Pt-Cu/NF, Pd-Cu/NF	Electrodeposition	0.05 M NO_3_-N in 0.5 M KCl	NH_3_ selectivity = 24%, removal rate = 93% at 0.1 A/cm^2^	[56]
Sn-Cu/Pt-NF	Electrodeposition	0.05 M NO_3_-N in 0.05 M KCl	NH_3_ selectivity = 39% and Sn enhanced selectivity towards N_2_	[57]
Cu/Ni	Electroless deposition	50 mg/L NO_3_-N in 0.1 M Na_2_SO_4_	High nitrate removal (97%) at 3 mA/cm^2^	[58]
Cu@Pd/PGE	Electrodeposition	10 mM in 0.1 M KOH	Nitrate removal of 46% at −1.0 V	This work
Cu@Pd/PGE	Electrodeposition	10 mM in 0.05 M Na_2_SO_4_	Nitrate removal of 59% at −1.3 V with high ammonia selectivity	This work

## Data Availability

The data supporting this article have been included as part of the Supplementary Information.

## Supplementary Materials

The following supporting information can be downloaded at: https://www.mdpi.com/article/10.3390/molecules30204062/s1, Figure S1: SEM images of copper nanostructures electrodeposited on pencil graphite electrode (a,b); Figure S2: OCP variation during the galvanic replacement reaction of palladium over Cu/PGE (a) and XPS deconvolution spectra of oxygen of Cu@Pd/PGE (b); Figure S3: Cyclic voltammograms obtained at different scan rates ranging from 10 to 100 mV/s in 0.5 M H_2_SO_4_ (a) and plot of current difference (ΔJ = J_a_ − J_c_) and scan rate at the potential 0.2 V (b); Figure S4: LSV profiles of Cu@Pd nanostructures with different copper deposition times (a), cycle life stability at −0.4 V vs RHE for 1000 potential cycles (b), amperometric stability of Cu@Pd/PGE electrodes at applied overpotential of 100 mV (c) and chronopotentiometric studies for Cu@Pd-7min towards HER (d): All the experiments were performed in 0.5 M H_2_SO_4_ at 50 mV/s scan rate; Figure S5: LSV curves of Cu/PGE in 0.1 M KOH in the absence and presence of nitrate (a) cyclic voltammograms of Cu/PGE in 10 mM of nitrate with varying scan rates from 10–100 mV/s scan rates (b) and corresponding linearity (c): All the experiments were performed in 0.1 M KOH with 10 mM of nitrate; Table S1: Parameters of fitting for the electrochemical impedance analysis over the Cu@Pd nanostructures using R(CR) equivalent circuit.
