# Supplementary material for: Rational Design of Cu@Pd Core–Shell Nanostructures via Galvanic Replacement for Dual Electrochemical Applications: Hydrogen Evolution and Nitrate Reduction Reactions
Source: Molecules. 2025 Oct 12;30(20):4062. doi: 10.3390/molecules30204062 (PMC12566024; doi:10.3390/molecules30204062)
Supplement: Supplementary file 1 [file molecules-30-04062-s001.zip › molecules-3902758-supplementary.pdf]

Rational Design of Cu@Pd Core–Shell Nanostructures via Galvanic Replacement for Dual Electrochemical Applications: Hydrogen Evolution and Nitrate Reduction Reactions

Bommireddy Naveen and Sang-Wha Lee *

Supporting Information

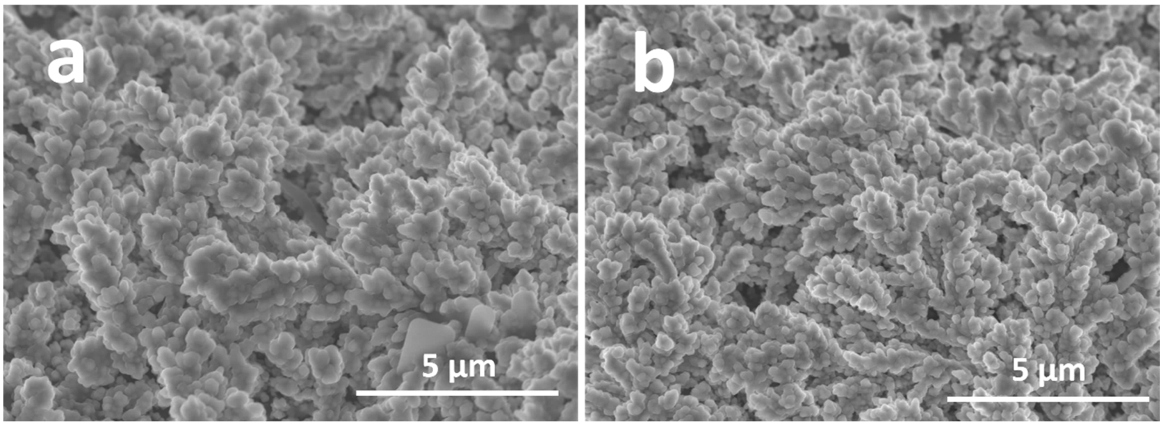

Figure S1: SEM images of copper nanostructures electrodeposited on pencil graphite electrode (a,b)

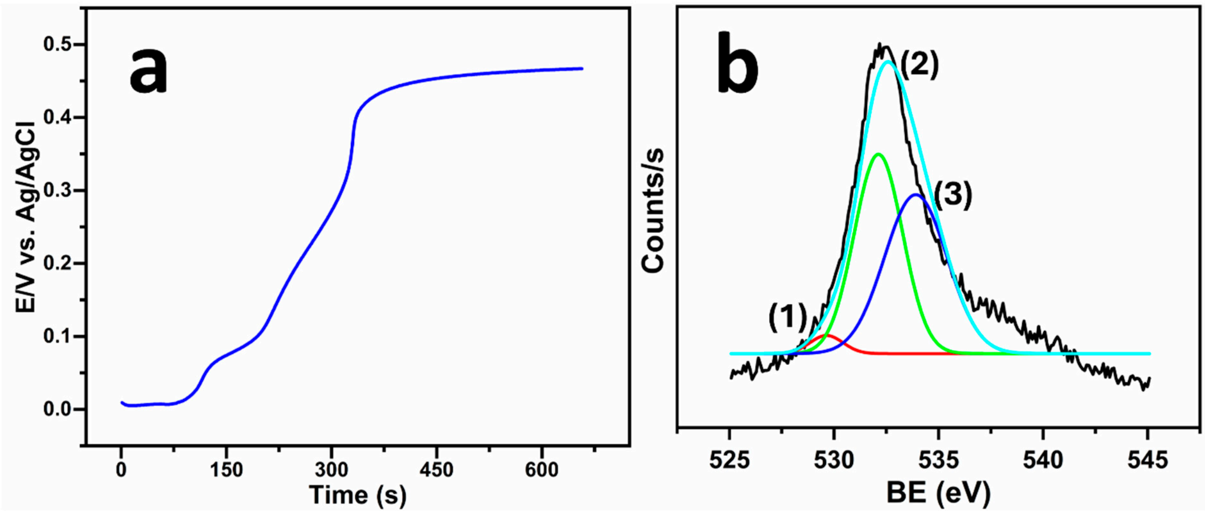

Figure S2: OCP variation during the galvanic replacement reaction of palladium over Cu/PGE (a) and XPS deconvolution spectra of oxygen of Cu@Pd/PGE (b)

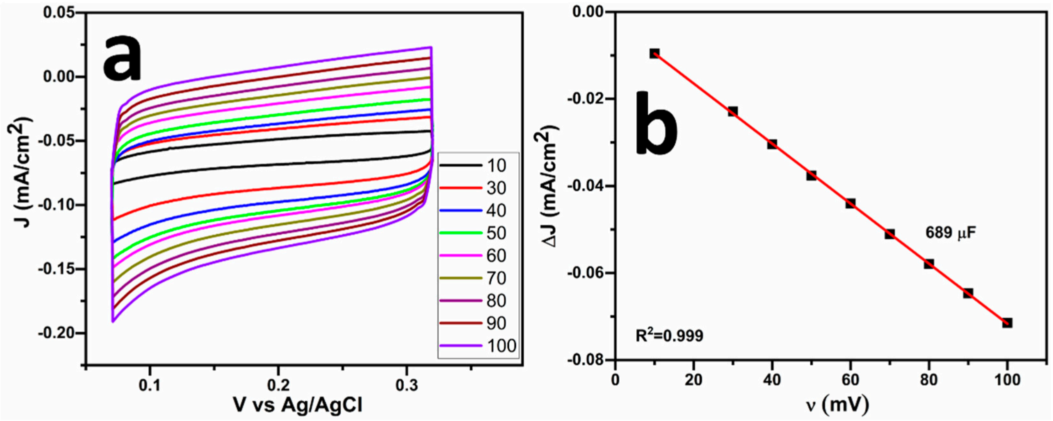

Figure S3: Cyclic voltammograms obtained at different scan rates ranging from 10 to 100 mV/s in 0.5 M H_2SO_4 (a) and plot of current difference ($\Delta J=J_a-J_c$) and scan rate at the potential 0.2 V (b)

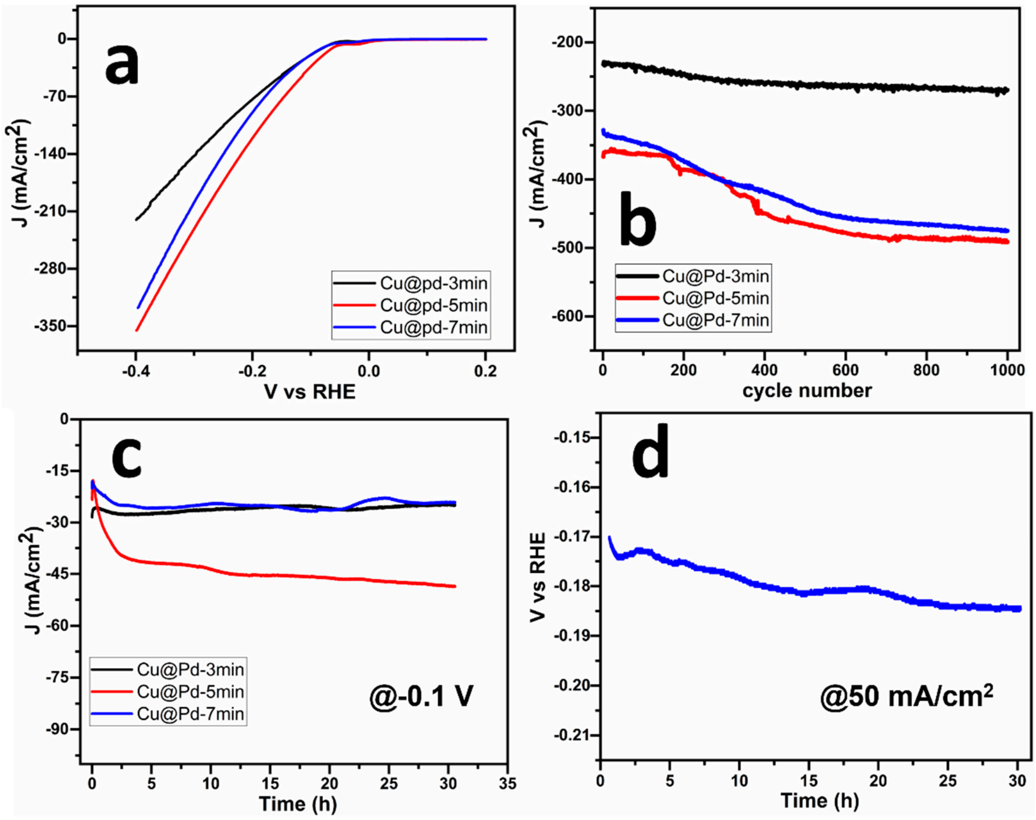

Figure S4: LSV profiles of Cu@Pd nanostructures with different copper deposition times (a), cycle life stability at -0.4 V vs RHE for 1000 potential cycles (b), amperometric stability of Cu@Pd/PGE electrodes at applied overpotential of 100 mV (c) and chronopotentiometric studies for Cu@Pd-7min towards HER (d): All the experiments were performed in 0.5 M H_2SO_4 at 50 mV/s scan rate.

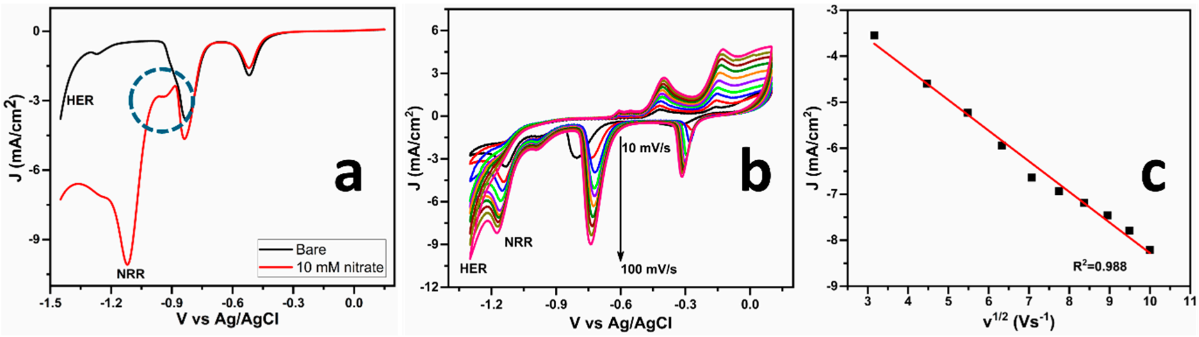

Figure S5: LSV curves of Cu/PGE in 0.1 M KOH in the absence and presence of nitrate (a) cyclic voltammograms of Cu/PGE in 10 mM of nitrate with varying scan rates from 10-100 mV/s scan rates (b) and corresponding linearity (c): All the experiments were performed in 0.1 M KOH with 10 mM of nitrate.

Table S1: Parameters of fitting for the electrochemical impedance analysis over the Cu@Pd nanostructures using R(CR) equivalent circuit.

Overpotential (η) Vs RHE	R_u (Ω)	C_{dl} (μF)	R_{ct} (Ω)
100 mV	2.4	3.75	50.4
50 mV	2.5	3.77	21.3
0 mV	2.5	4.05	9.9
-50 mV	2.5	6.17	6.2
-100 mV	2.6	5.43	4.5
-150 mV	2.6	5.28	3.9
